# Ethnic Bullying Victimization in Italy: The Role of Acculturation Orientation for Ethnic Minority Adolescents With Differing Citizenship Statuses

**DOI:** 10.3389/fpsyg.2020.00499

**Published:** 2020-03-19

**Authors:** Benedetta Emanuela Palladino, Maria Rosaria Nappa, Valentina Zambuto, Ersilia Menesini

**Affiliations:** Department of Education, Languages, Intercultures, Literatures and Psychology, University of Florence, Italy

**Keywords:** ethnic bullying, victimization, citizenship status, immigrant status, acculturation orientation, ethnic minority

## Abstract

This study examines the role of acculturation orientation toward home and host countries in ethnic bullying victimization, by considering citizenship status and taking into account social withdrawal. Within a larger school project with middle and high school students, we analyzed data on 252 adolescents from immigrant backgrounds: 71 born abroad without Italian citizenship (Males = 71.4%; mean age = 13.98, *SD* = 1.7); 96 born in Italy to immigrant parents and without Italian citizenship (Males = 58.3%; mean age = 13.26, *SD* = 1.6); and 85 Italian citizens born in Italy with an immigrant parent (Males = 54.7%; mean age = 13.12, *SD* = 1.5). At the univariate level we found that the group of adolescents born abroad with foreign parents showed significantly higher levels of ethnic victimization compared to the group of adolescents born in Italy with an Italian parent. The latter also reported a significantly higher mean in Acculturation Orientation toward their Host Country (i.e., Italy) compared to the other two groups. Looking at the processes working within each group, we found differences in the patterns of association between acculturation orientation and ethnic bullying victimization. Specifically, we found a significant and positive association between acculturation orientation toward the home country and ethnic victimization in the two groups of adolescents born in Italy, while acculturation orientation toward the host country seems to be a protective factor only for adolescents with Italian citizenship. Acculturation orientation does not play any role in ethnic victimization for the first generation of immigrants, while for this group we found a stronger positive effect of Social Withdrawal. Citizenship status appears to be a good indicator of belonging to an ethnic minority group with a background of immigration: it seems to catch specific processes in ethnic bullying victimization.

## Introduction

Research shows that children with non-normative behaviors or physical characteristics that differ from the majority of the peer group are at increased risk of victimization (Juvonen et al., 2003). Juvonen and Graham (Juvonen and Graham, 2014) describe a type of bullying based on students’ prejudice – toward the race, ethnicity or national origin, disability, sexual orientation or gender identity, or religion of their peers. This prejudicial bullying is the expression of racism, sexism, religious intolerance, and sexual prejudice (Goodboy et al., 2016) and is defined *prejudice-related bullying* or *bias-based bullying* (Menesini and Salmivalli, 2017). As traditional bullying, *bias-based bullying* can have different forms: verbal, physical attacks, or direct and indirect social exclusion (Cooley et al., 2016; Brenick and Halgunseth, 2017). Due to the cultural content, the intensity of this kind of bullying may vary across contexts (Durkin et al., 2012). When prejudice against a specific characteristic is strongly rooted in a particular social context, perpetrators often do not perceive their actions as damaging or offensive (Sue, 2017), and bystanders tend not to intervene to defend peer victims (Palmer and Abbott, 2018).

Among the forms of prejudice-related bullying, bullying that targets the student’s cultural background or identity is defined as *ethnic bullying* (McKenney et al., 2006). It includes overt (e.g., racial slurs, derogatory references to culturally specific customs etc.) and covert harassment (e.g., social exclusions) and can be extended to the student’s immigrant status or the family’s background of immigration (Scherr and Larson, 2009). Just as traditional bullying, ethnic bullying is characterized by the intention to hurt, systematic aggressions over time, and the imbalance of power between the victim and the aggressor (Farrington, 1993; Olweus, 1993).

Within an individual by context framework, it is important to look at the minority children while also referring to a broad ecological interactional model (Garcìa Coll et al., 1996) where we can better understand a social phenomenon such as ethnic bullying. What youth do in terms of acculturation orientation toward both their home and host countries have a relevant impact on the social peer environment, but only a few studies have been carried out on this specific relation, and with mixed results (Kvernmo and Heyerdahl, 2003; Romero and Roberts, 2003; Roberts and Ali, 2013). At the same time, it is important to understand the effects of this variable on ethnic bullying by taking into account the effects of other processes that work at the individual level in the social adaptation (i.e., social withdrawal).

### Race, Ethnicity, and Immigrant Status

A growing body of research is focused on the role of race, ethnicity and immigrant status on victimization in the school context (Juvonen et al., 2006; Juvonen and Graham, 2014), with conflicting results. Some studies report that ethnic minority groups were more victimized than ethnic majority groups (Nansel et al., 2001; Graham and Juvonen, 2002). Conversely, a recent meta-analysis that examined studies on prevalence of bullying victimization among ethnic majority youth, underlined that ethnicity was not strongly and unequivocally associated with victimization (Vitoroulis and Vaillancourt, 2015). The authors coded majority vs. minority by observing both visible (e.g., African American in the US) and non-visible ethnic minority groups (e.g., Former Yugoslavian in Austria), and using immigrant status and language as a proxy indicator of ethnic status. Conversely, another systematic review (Pottie et al., 2015) showed that immigrant adolescents born abroad seem to experience a higher rate of bullying and peer aggression compared to their native counterparts. In considering these latest findings (Pottie et al., 2015), we should take into account that they mainly considered US studies, as only 3 out of 18 were from European countries.

Belonging to an ethnic minority group and being an immigrant might co-occur in some context and are commonly used as proxy indicators of ethnic status, albeit with a great variability across countries (Strohmeier et al., 2011). For example, most of the studies on the victimization of minority groups conducted in the US consider the race or ethnicity of students, because the racial/ethnic makeup of schools is complex and diverse (Hanish and Guerra, 2000; Graham and Juvonen, 2002; Maynard et al., 2016). On the contrary, European studies on discrimination and bullying in school are predominantly focused on immigrant status: the vast majority of students in schools are Caucasian, thus different groups are categorized in relation to nationality, geographical origin, and citizenship (Motti-Stefanidi et al., 2008; Zaiceva and Zimmermann, 2008; Thomas, 2012). The history of immigration, ethnic diversity, and status of ethnic minorities varies consistently between North American countries and European countries, as well as across countries within Europe, with a huge variation in levels of negative attitudes toward specific ethnic groups and acceptance of multiculturalism.

Within the studies focused on the European population, there is still much variability in findings on the differences in victimization between immigrant students and their native counterparts. While no differences between natives and immigrant students have been reported in studies on general victimization (Strohmeier et al., 2008; Fandrem et al., 2009; Strohmeier and Spiel, 2012), other studies focusing specifically on ethnic attacks underlined a higher level of racist victimization (e.g., racist insults or name-calling) in immigrant students as compared to native counterparts (Jasinskaja-Lahti and Liebkind, 2001; Verkuyten and Thijs, 2002; Monks et al., 2008). Immigrant youths could evoke negative and discriminatory reactions in native groups due to their cultural differences (McDonald et al., 2012). Therefore, it is possible to hypothesize that these inconsistent results could be due to the specific outcome taken into consideration: general bullying victimization vs. specific subtypes of bullying related to ethnic bullying.

Looking at the specific association between immigrant status and bullying victimization, the mixed findings suggest that the relationship could also be moderated by contextual and individual factors. Moving from the pioneer study of Thomas and Znaniecki (1918-1920), literature describing immigrants as a heterogeneous group use often the immigrants’ generations variable (first, second, and third generation) as a proxy indicator (Peskin et al., 2006; Strohmeier et al., 2011; Vitoroulis and Georgiades, 2017). Commonly, the first generation refers to immigrants born abroad and the second generation defines people born in the host country with foreign-born parents. The third generation refers to individuals with both parents born in the host country but at least one foreign-born grandparent. In general, it has been demonstrated that there are substantial differences between different generations of immigrant groups (Christmas and Barker, 2014). First generation immigrants deal with the challenge of resettlement in a host country: adolescents enter a new community and a new school while also usually acquiring new language skills (Acevedo-Garcìa et al., 2012). On the other hand, second generation adolescents have spent their entire life in the host country with linguistic competences comparable to their native counterparts and familiarity with the host country culture (Alba and Nee, 2009; Plenty and Jonsson, 2017), but they belong from minority status families.

#### The Specificity of the Italian Context

Italy started to grapple with immigration issues in recent years, while historically it was considered a country affected by emigration processes. In 2017, in the Italian context, the number of foreign children and adolescents was about 1 million (International Organization for Migration [IOM], 2018), including foreign born children (first generation of immigrants) and children born in Italy with foreign parents who have not yet obtained citizenship (second generation immigrants). 9.2% of all students enrolled in Italian schools do not have Italian citizenship (77% of them are non-EU citizens). Forty six percent of all foreign students are enrolled in primary schools, a quarter in secondary-lower schools, and 29% in secondary-higher schools (MIUR, 2018).

The current law in Italy is based on the *ius sanguinis* principle (Law n. 91, 1992). Specifically, people acquire Italian citizenship if they have blood ties with an Italian citizen. For example, children can acquire Italian citizenship by birth if the father or the mother already have it. Differently from other countries (e.g.,USA follows the *ius soli* principle), being born in the country does not lead to the automatic acquisition of citizenship if both parents are foreign citizens. When children turn 18 years old they can formally request citizenship, but only under certain conditions (e.g., having resided in Italy for their previous entire life; the parents must have applied for resident status upon birth, etc.). In 2016 and 2017 in Italy legislation was proposed to reform the current law, introducing the *ius soli* principle. This took center stage in Italy’s political debate, polarizing the country’s public opinion (Tintori, 2018). In December 2017 the campaign to reform ius soli was definitively abandoned and anti-immigration supporters started to play a central role in the political debate. At present, adolescents born and raised in Italy by immigrant parents (i.e., second-generation immigrants) don’t have the right to become Italian citizens until they come of age.

Looking at the relation between the generation of immigrants, adaptation and bullying, a recent study in Italy (Borraccino et al., 2018) found that students from Eastern European and non-Western/non-European countries had a higher occurrence of health complaints and the highest risk of reporting low life satisfaction, which increased among the second generation. While they found that the risk of reporting bullying behaviors was higher in first-generation immigrants and decreased in the second generation, independently of ethnic background, results about specific ethnic bullying victimization were not reported. Literature on this topic is still scarce in Italy.

### The Role of Acculturation Orientation

The relationship between ethnicity, race and/or immigrant generation, and bullying victimization could be influenced by variables working both at the contextual and at the individual level that interact in the social dynamics of bullying. Some studies underline the importance of the societal level (Verkuyten and Thijs, 2002; Verkuyten and Yildiz, 2007) for adaptation, while at the school and class levels strong attention has been given to the proportion of minority or majority groups and to the conflict that this balance can generate (Vervoort et al., 2010; Agirdag et al., 2011; Schumann et al., 2013; Jansen and Leukfeldt, 2016; Lanza et al., 2018). Looking at the individual level, the role of acculturation orientation toward both host and home country may play an important role in immigrant wellbeing and adaptation (Berry, 1997, 2005; Phalet and Schönpflug, 2001).

Acculturation has been defined as “*a group of phenomena which result when groups of individuals having different cultures come into continuous first-hand contact, with subsequent changes in the original cultural patterns of either or both groups*” (Redfield et al., 1936). Berry (1997, 2005) introduced the acculturation orientation framework to study how different generations of immigrants adapt in a new cultural context. The author describes a multidimensional process characterized by two opposite tendencies: preserving their own cultural identity and identifying with the culture of the host country. Berry distinguishes four different acculturation orientations (1997): integration – acculturation toward both own origin country and host country cultures, assimilation – acculturation toward the host country culture, separation – exclusive maintenance of own cultural identity, marginalization – inobservance of both cultures.

The adoption of one or another of these acculturation strategies has been associated with lower or higher levels of adaptation (Kvernmo and Heyerdahl, 2003) while few studies looked at the link with bullying victimization. Generally, it can be deduced that integration is the most adaptive approach (Schwartz et al., 2010). Smokowski et al. (2009), in a review underlined that the link between acculturation orientation and violence in adolescents belonging to minority groups depends on the specific ethnic group identity or culture of origin. Another review about acculturation models highlights the central role of individuals’ processes, such as the coping strategies of migrants and different ethnic populations (Kuo, 2014). The transmission of cultural heritage by family represents a protective factor for immigrant adolescent wellbeing (Juang and Alvarez, 2010). On the other hand, the different levels of identification with the dominant culture could influence the social environment that can foster bullying (Romero and Roberts, 2003). Mendez et al. (2012) show that the immigrant status is associated to bullying victimization even within the same ethnic group when there is a different level of acculturation toward the host culture. Moreover, Messinger et al. (2012) observing a Spanish sample, found that immigrant youths that experience difficulties with the acculturation process – defined as “acculturation stress”- tend to be involved in bullying, both as victims and bullies. Roberts and Ali (2013) with their research on ethnic minority children in Australia, found that students who adopted an integration acculturation orientation experienced less victimization in comparison to peers that adopted an assimilation strategy.

### The Impact of Social Withdrawal

The sole presence of ethnic or immigrant diversity at school can establish the prerequisite of a power imbalance among different groups (Scherr and Larson, 2009). Social minorities that are ethnically, culturally, and/or linguistically different from the majority group can be targeted because of their marginal social status (Hawley and Williford, 2015; Yeager et al., 2015). At the same time, this relation could be direct or indirect: since other intrapersonal and interpersonal variables, such as social withdrawal (Hodges and Perry, 1999), could have a buffer effect (Maynard et al., 2016).

Different studies have shown that social withdrawal *per se* is associated with a higher risk of peer victimization (Juvonen, 1991; Wood et al., 2002). This construct has been identified as an umbrella term including shyness, behavioral inhibition, isolation, social reticence, passivity. It is correlated to different causes (e.g., temperamental disposition, peer rejection, psychological maladaptation) (Rubin and Coplan, 2004; Rubin et al., 2009). Being isolated and marginalized in class, shy students could represent easy targets for violence and bullying (Hodges and Perry, 1999; Hubbard et al., 2001; Rubin et al., 2006). This view is also consistent with Perry and colleagues’ definition of “passive victims” (Perry et al., 1988). Furthermore, withdrawn children and adolescents may be rejected by their peer group, which in turn leads to a higher risk of peer victimization (Dill et al., 2004). Finally, socially withdrawn children who have been rejected by their peers on the grounds that their behavior differs to age-specific norms and expectations for social interaction (Rubin et al., 2006), reinforce their own marginal position on the group. Several studies highlight that social withdrawal could be an additional factor of vulnerability for immigrant students. On the other hand, social withdrawal may also be a possible outcome of migration and cultural diversity: it has been demonstrated that culture shock and culture conflict could predispose some immigrants to withdraw socially, and develop a sense of despair and alienation (Bhugra and Ayonrinde, 2004; Messinger et al., 2012).

### The Current Study

Belonging to a specific ethnic minority group (Smokowski et al., 2009) and the status of immigrant (Mendez et al., 2012) are proxy indicators of the ethnic status and both seem to be related differently to bullying victimization (Pottie et al., 2015; Vitoroulis and Vaillancourt, 2015). Indeed, they could co-occur, but they are not the same thing (Strohmeier et al., 2011). Besides, they are influenced by contextual factors such as the specific country context, its current law about the acquisition of the citizenship and its recent history of immigration. At the same time, shifting the focus between the general bullying phenomenon or the specific ethnic subtype – i.e., ethnic bullying, seems to have an impact on the link between ethnic status and victimization (Jasinskaja-Lahti and Liebkind, 2001; Verkuyten and Thijs, 2002; Monks et al., 2008; Strohmeier et al., 2008; Fandrem et al., 2009; Strohmeier and Spiel, 2012).

To understand the link between acculturation orientation both toward one’s home and host country and ethnic bullying victimization, the present study uses a specific proxy indicator of belonging to an ethnic minority: the *citizenship status* that is highly relevant in Italy.

Specifically, we use a composite measure that takes into account both the participants’ and their parents’ country of origin. This allows us to define whether the participants are eligible or not for Italian citizenship. We focalized our attention on citizenship status, since in our country it could be a more accurate proxy to capture differences in youth population. It considers first- and second-generation immigrants, but also nuances derived from the host country’s perception of “who you are.” Not only contingent research which categorized heterogeneous participants, but also previous studies have suggested that naturalization, and thus citizenship to the country of residence, is a prerequisite for successful acculturation, and particularly for integration.

Citizenship can be viewed as cultural recognition -at the national level- of the ingroup/outgroup belonging, although this can be moderated by individual variables such as social withdrawal. This variable, in fact, can reduce the process of acculturation and socialization of immigrant children and can have a cumulative effect with general bullying, often directed toward shy and withdrawn peers (Hodges and Perry, 1999; Hubbard et al., 2001; Rubin et al., 2006).

Summarizing, the aim of the current study is twofold: analyzing the role played by individual variables, namely two different aspects of social adaptation (i.e., acculturation orientation and social withdrawal) in ethnic bullying victimization; testing if the citizenship status can be a useful proxy to capture differences in the phenomena analyzed and in the processes that lead into ethnic bullying victimization.

First, we hypothesized that students born abroad and students born in Italy with foreign parents (without Italian citizenship), are at a higher risk of ethnic bullying victimization (Strohmeier et al., 2011; Pottie et al., 2015) compared to students with Italian citizenship. Second, we hypothesized that acculturation toward Italy and toward one’s home country may play a relevant role in bullying (Roberts and Ali, 2013) along with citizenship status. Considering the consistent findings in the literature, we hypothesize that social withdrawal is associated with a higher risk of ethnic bullying victimization both because it is an additional factor of vulnerability for immigrants and it is a possible outcome of migration and cultural diversity.

## Materials and Methods

### Participants

Within a larger school project (*N* = 1366; grades 7th to10th; Male = 71.4%; mean age = 13.98, *SD* = 1.7, MIN = 11, MAX = 18; middle school = 59.9%) carried out in the 2016/2017 school year, we collected data of about 252 adolescents with an immigrant background attending middle (*N* = 13) and high (*N* = 10; 1 lyceum, 7 technical and 2 vocational) schools in Tuscany. We defined the *citizenship status* by looking at three questions about the country of origin of the participants, their fathers and their mothers (“Where were you born?”; “Where was your mother born?”; “Where was your father born?”). The first group (A) is composed of 71 *adolescents born abroad* with foreign parents (Males = 71.4%; mean age = 13.98, *SD* = 1.7, MIN = 11, MAX = 17; middle school = 47.1%): they are first generation immigrants and they do not have Italian citizenship. The second group (B) is composed of 96 *adolescents born in Italy to foreign parents* (Males = 58.3%; mean age = 13.26, SD = 1.6, MIN = 11, MAX = 17; middle school = 53.1%): they are second generation immigrants, but they do not have Italian citizenship – they can request it when they turn 18 years old. Finally, the third group (C) is composed of 85 *adolescents born in Italy to an Italian parent* and an immigrant one (Males = 54.7%; mean age = 13.12, *SD* = 1.5, MIN = 11, MAX = 18; middle school = 61.6%): they spent the same amount of time in Italy compared to the second group, but they have had Italian citizenship since birth. In Table 1 the countries of origin of the participants and their parents are reported.

**TABLE 1:** The countries of origins of the participants and their parents: frequencies and percentages on the total.

	Adolescents		Mothers		Fathers	
			
	n	%	n	%	n	%
Albania	17	6.7	57	22.6	56	22.2
China	2	0.8	5	2.0	5	2.0
Philippines	2	0.8	8	3.2	4	1.6
Morocco	9	3.6	25	9.9	26	10.3
Peru	4	1.6	6	2.4	5	2.0
Poland	1	0.4	5	2.0	1	0.4
Romania	20	7.9	35	13.9	34	13.5
Sri Lanka	2	0.8	6	2.4	7	2.8
Switzerland	0	0.0	5	2.0	5	2.0
Tunisia	0	0.0	1	0.4	4	1.6
Ukraine	1	0.4	5	2.0	2	0.8
Italy	181	71.8	30	11.9	55	21.8
Other countries	13	5.2	64	25.4	48	19.0
Total	252	100%	252	100%	252	100%

### Measures

Adaptation of the florence victimization and bullying scales (Palladino et al., 2016). In order to measure the specificity of ethnic bullying, we developed an additional subscale for both perpetration of ethnic bullying (4 items) and victimization (4 items). After showing a general definition of bullying, we ask how often in the past couple of months respondents have experienced particular attacks about physical (i.e., “I have been beaten…”), verbal (i.e., “Someone made fun of me.”) and indirect forms (i.e., “Rumours about me.” and “I have been excluded…”) because of my culture or country of origin.” We developed 8 items rated by participants on a 5-point Likert scale from “never” to “several times a week”: each one was followed by the ethnic related specification. For the purpose of the present study we only used data about ethnic bullying victimization (4 items). The scale showed good reliability, assessed by Cronbach’s alpha (α = 0.73). The Confirmatory Factor Analysis (CFA) also showed good fit indexes (χ^2^ = 2.050, *DF* = 2, *p* = 0.36; RMSEA = 0.009, 90% CI [0.000–0.118]; CFI = 0.998; SRMR = 0.039; factors loadings ranged from 0.386 to 0.743; for fit indexes cut-offs and mean see Hu and Bentler (1999). For the following analyses the saved factor scores were used.

The brief acculturation orientation scale (Demes and Geeraert, 2014) independently assesses acculturation orientation toward the home country and the host country (Italy in our case). Four central indicators of acculturation orientation (i.e., the value of cultural friendships, traditions, characteristics, and actions) are presented twice, once for the home country and again for the hosting country (e.g., “It is important for me to have [home country] [host country] friends”). Participants are asked to rate their agreement on a 7-point Likert scale, ranging from “strongly disagree” to “strongly agree.” The scales showed good reliability, assessed by Cronbach’s alpha (acculturation orientation toward: home country, α = 0.77; host country, α = 0.83). The CFA of the bi-dimensional model also showed good fit indexes (χ^2^ = 22.249, *DF* = 18, *p* = 0.22; RMSEA = 0.032, 90% CI [0.000- 0.071]; CFI = 0.990; SRMR = 0.047; factors loadings ranged from 0.362 to 0.917, a covariance between residuals of the two subscales was included following the Modification Indexes; for fit indexes cut-offs and mean see Hu and Bentler (1999). For the analyses we used the saved factor scores.

The social withdrawal scale is derived from the Youth Self Report (Achenbach, 1991). It is composed by 6 items (e.g., “I am shy,” “I prefer to be alone rather than with others”): the behaviors are rated by participants on a 3-point scale (i.e., “Not true”, “Somewhat or sometimes true” and “Very true or often true”) based on the preceding 6-months. The scale showed good reliability, assessed by Cronbach’s alpha (α = 0.73) and the Confirmatory Factor Analysis (CFA) also showed good fit indexes (χ^2^ = 8.370, *DF* = 8, *p* = 0.50; RMSEA = 0.000, 90% CI [0.000–0.070]; CFI = 1.000; SRMR = 0.027) For the analyses we used the saved factor scores.

### Plan of Analysis

We analyzed data in two steps. Firstly, we looked at possible differences at the univariate level in the variables of interest. Using ANOVAs, we tested for differences between the three groups in ethnic bullying victimization, acculturation orientation toward both home and host countries and social withdrawal. Effect size was evaluated by mean of eta squared (above 0.02 = small, above 0.13 = medium, above 0.26 = large; Cohen, 1988) and significant *post hoc* analyses were reported using Tukey’s test. As a second step, we looked at the differences in the processes across the three groups. Using multiple group regression analyses, we tested a model in which ethnic bullying victimization is regressed on acculturation orientation toward both home and host countries, controlling for the effect of social withdrawal. The analyses were conducted with SPSS (Ibm Corp, 2017) and Mplus 7.0 (Muthén and Muthén, 1998-2012).

## Results

STEP 1 – At the univariate level (see Table 2) we found that group (A) – adolescents born abroad with foreign parents, showed a significantly higher level of ethnic victimization compared to group (C) – adolescents born in Italy with an Italian parent [*F*_(__2_,_241__)_ = 4.041, *p* = 0.019; η^2^ = 0.03, small effect size]. For Acculturation Orientation toward Host Country (Italy) we found that the group (C) – adolescents born in Italy with an Italian parent- reported a significantly higher mean in this variable compared to the other two groups [*F*_(__2_,_191__)_ = 7.317, *p* = 0.001; η^2^ = 0.07, small effect size]. For Acculturation Orientation toward Home Country [*F*_(__2_,_191__)_ = 1.223, *p* = 0.297] and Social Withdrawal [*F*_(__2_,_230__)_ = 1.439, *p* = 0.239] no significant differences were found.

**TABLE 2:** Descriptive Statistics and ANOVAs results.

	Adolescents born abroad with foreign parents (A)		Adolescents born in Italy with foreign parents (B)		Adolescents born in Italy with an Italian parent (C)		ANOVAs	*Post hoc* results
			
	Mean	*SD*	Mean	*SD*	Mean	*SD*		
Ethnic bullying victimization	0.08	0.48	0.04	0.42	–0.10	0.39	***F*_(__2_,_241__)_ = 4.041, *p* = 0.019; η^2^ = 0.03**	(A) vs. (C)
Acculturation Orientation toward Home Country	–0.14	1.09	0.09	0.81	0.04	0.84	*F*_(__2_,_191__)_ = 1.223, *p* = 0.297	–
Acculturation Orientation toward Host Country (Italy)	–0.07	0.52	–0.08	0.52	0.25	0.40	***F*_(__2_,_191__)_ = 7.317, *p* = 0.001; η^2^ = 0.07**	(A) (B) vs. (C)
Social Withdrawal	0.07	0.42	–0.01	0.36	–0.04	0.37	*F*_(__2_,_230__)_ = 1.439, *p* = 0.239	–

**In bold the significant differences (p < 0.05).**

STEP 2 – Looking at the processes working within each group, we found differences in the patterns of association between acculturation orientation and ethnic bullying victimization (in Table 3 the Multiple Group Regression Analyses results are reported). Specifically, in the first group (A) – Adolescents born abroad with foreign parents, we did not find any significant association between acculturation orientation toward home and host country and the outcome (respectively, β = 0.015, S.E. = 0.119, *p* = 0.902; β = -0.018, S.E. = 0.153, *p* = 0.906). Conversely, in the second generation immigrants, we found a significant positive association between acculturation orientation toward the home country and ethnic bullying victimization. This was true for the second group (B) – Adolescents born in Italy to foreign parents (β = 0.178, S.E. = 0.067, *p* = 0.007) as well as for the third (C) group -Adolescents born in Italy with an Italian parent (β = 0.238, S.E. = 0.108, *p* = 0.025). Only in the third group (C) -Adolescents born in Italy with an Italian parent, we also found a significant negative association between Acculturation Orientation toward the Host Country (Italy) and the outcome variable (β = -0.422, S.E. = 0.162, *p* = 0.009).

**TABLE 3:** Multiple group regression analyses predicting ethnic bullying victimization from acculturation orientation toward home and host country, controlling for social withdrawal.

	Ethnic bullying victimization								

	Adolescents born abroad with foreign parents (A)			Adolescents born in Italy with foreign parents (B)			Adolescents born in Italy with an Italian parent (C)		
			
	β	S.E.	*p*	β	S.E.	*p*	β	S.E.	*p*
Acculturation Orientation toward Home Country	0.015	0.119	0.902	**0.178**	**0.067**	**0.007**	**0.238**	**0.108**	**0.025**
Acculturation Orientation toward Host Country (Italy)	−0.018	0.153	0.906	−0.139	0.078	0.076	**−0.422**	**0.162**	**0.009**
Social Withdrawal	**0.483**	**0.111**	**<0.001**	**0.306**	**0.085**	***p* < 0.001**	**0.425**	**0.151**	**0.005**
Total *R*^2^	**0.24 (*p* < 0.001)**			**0.13 (*p* < 0.001)**			**0.27 (*p* < 0.001)**		

**In bold the significant patterns (p < 0.05).**

In all the models the effect of Social Withdrawal was significant: the association was positive and significant in each group [group (A) β = 0.483, S.E. = 0.111, *p* ≤ 0.001; group (B) β = 0.306, S.E. = 0.085, *p* < 0.001; group (C) β = 0.425, S.E. = 0.151, *p* = 0.005]. Therefore, we can say that the role of acculturation toward the home and host country was significantly above and beyond personal characteristics of social withdrawal, but future studies are needed to better understand how these variables can interact together.

The overall variance explained by the models were respectively 24% (group A)- 13% (group B) and 27% (group C).

## Discussion

Nowadays, ethnic bullying, a type of bias-based bullying that targets students’ ethnic background or cultural identity, is getting more and more attention because of the consistency of migration processes, the population diversity at school and the consequences of this problem for the youth involved. The current study improves our knowledge of ethnic bullying victimization, focusing on the role played by individual variables, such as acculturation orientation and social withdrawal, in the group dynamics. At the same time, we also tested if the citizenship status can be a useful proxy to capture differences in the phenomena and in the processes that lead into ethnic bullying victimization.

Looking at the country of origin of the participants and their parents’, our sample is characterized by a great variability in ethnic backgrounds. This variability reflects the history of different fluxes of immigration and prevented us from grouping the participants simply by their country of origin. To deepen the indicator of being an ethnic minority, we focalized our attention on citizenship status, which takes into account not only first and second generation immigrants, but also additional nuances related to recognition of who the immigrants are, in that country. Proxy indicators of ethnic status used in the literature varies. Race is widely used, especially in the US, because the racial/ethnic makeup of schools are complex and various (Hanish and Guerra, 2000; Graham and Juvonen, 2002; Maynard et al., 2016). Being an immigrant – first or second generation, is a preferred indicator in European countries because the vast majority of students in schools are Caucasian, thus they are categorized regarding nationality or geographical origin (Motti-Stefanidi et al., 2008; Zaiceva and Zimmermann, 2008; Thomas, 2012). Additionally to geographical background origins, previous studies have suggested that naturalization, and thus citizenship to the country of residence, is a prerequisite for successful acculturation and integration (Maehler et al., 2019). The results highlight the role of citizenship status particularly the differences of the three groups along with specific processes in ethnic bullying victimization as being a good indicator of belonging to an ethnic minority group.

In the present study we found, at a univariate level, that adolescents born abroad without Italian citizenship show higher levels of ethnic victimization compared to adolescents born in Italy with an Italian parent who, by virtue of bloodline have Italian citizenship. This result confirmed previous studies that focalize their attention on racist/ethnic victimization in immigrant youth (Jasinskaja-Lahti and Liebkind, 2001; Verkuyten and Thijs, 2002; Monks et al., 2008). Immigrant students are explicitly attacked due to their cultural characteristics (e.g., language, food, clothes, etc.). A first-generation immigrant’s cultural background could differ significantly from the majority group (Acevedo-Garcìa et al., 2012). Specifically, foreign-born adolescents are perceived more likely as outgroup members (Phinney et al., 2000) and are at higher risk of being victimized than their second and third generation peers (Strohmeier et al., 2011; Pottie et al., 2015). At the same time, we should also notice that the group of adolescents born in Italy from parents born abroad does not differ from the other groups, confirming that the citizenship categorization was able to detect specificities that can be lost focusing only on the first and second generation classification of participants.

The other difference we found at the univariate level was related to acculturation orientation toward Italy, the host country. Specifically, adolescents born in Italy with an Italian parent show higher values in this acculturation process toward the hosting country. It is quite understandable considering that probably they also have Italian relatives that can foster this process. At the same time no difference was found in relation to acculturation orientation toward the country of origin. This finding underlies the importance of roots and boundaries for all the participants regardless to the citizenship status.

Looking at the processes associated with ethnic bullying victimization, we found that acculturation orientation toward one’s home country represents a risk factor for adolescents born in Italy with an immigrant background, i.e., with one or two parents born abroad. A strong or accentuated acculturation orientation toward one’s home country, defined as separation (Berry, 1997), represents a risk factor for immigrant youth and ethnic minority adolescents (Kvernmo and Heyerdahl, 2003): it has already been demonstrated that separation and marginalization acculturation orientation strategies are associated with behavioral problems. They could in turn predict victimization experiences. Identification with a foreign culture, and exclusive friendships with peers that have immigrant backgrounds can reinforce a minority status that is the core of bias-bullying and ethnic bullying phenomena (McKenney et al., 2006; Menesini and Salmivalli, 2017).

For adolescents born abroad, that have a first hand history of immigration, acculturation orientation toward one’s home country does not play a role in ethnic bullying victimization. Probably, visible diversity factors (Alba and Nee, 2009; Strohmeier and Spiel, 2012), such as poorer language competence, unawareness of cultural traditions or few social boundaries, for example with school peers, play a stronger role in determining their minority status. Spending more time in the new country increases immigrant adolescents’ language competence, cultural knowledge, and promotes the adoption of new sociocultural behavior: immigrant adolescents’ cultural identity becomes more similar to that of a native-born (Azzolini et al., 2012). Looking at the effects of social withdrawal in this group seems more relevant than the role played by this individual level characteristic. Being shy and withdrawn can affect the process of acculturation and in turn increase ethnic victimization. In fact, socially withdrawn children and adolescents may have difficulty making friends (Pedersen et al., 2007) and this factor can have an additive effect on social relationships and integration (Rubin et al., 2006).

In our sample, acculturation toward the host county represents a protective factor only for students who were born in Italy and have an Italian parent – the group of adolescents with Italian citizenship, the group that expresses a higher level of acculturation orientation toward Italy. Conversely, this variable is not a significant protective factor for adolescents who do not have Italian citizenship: not for the first-generation immigrants, nor for those who have spent their entire life in Italy. Titzmann et al. (2008) underline how the transition to a new country implies a reorganization of cultural values, and behavioral systems. During this process, protective factors are less powerful in shielding a person from negative outcomes, specifically for those who live through the initial phases of immigration. Third generation immigrants are more likely to adopt their host country’s cultural patterns: this involves the development of affiliations between minority members and majority groups (Alba and Nee, 2009) and more supportive peer relationships (Strohmeier and Spiel, 2012). At this level, the acculturation process can be helpful to avoid experiences of ethnic victimization (Birman and Trickett, 2001). This process contributes to explain how third generation immigrants report high level of school safety (Peguero, 2008).

Another explanation could be related to the status of citizenship. In the socio-cultural context, having citizenship could be not only connected to political benefits, but also to integration at an institutional level and how people are recognized and perceived by the majority group. Ethnic bullying, as a bias-based phenomenon, is strongly related to specific socio-cultural contexts (Sue, 2017). Bullying directed at ethnic minorities or immigrant youths could reflect societal prejudice and become part of the peer culture at school (Scherr and Larson, 2009). Although acculturation orientation is more connected to the individual sphere (Kuo, 2014), it regards the adaptation process in the social context. Through this group perspective we can understand how acculturation orientation toward one’s host country does not have a protective role for adolescents born in Italy to foreign parents, and how acculturation orientation, both toward one’ home and host country, does not have any impact on the risk of victimization for adolescents born abroad.

In every group, social withdrawal is always associated with ethnic victimization. We included this variable in our models in order to control for other processes working at the individual level that affect social relationships. In fact, social withdrawal represents one of the strongest correlations and consequences of peer rejection during middle childhood and adolescence (Deater-Deckard, 2001; Dill et al., 2004; Rubin et al., 2006). Regarding the first generation of immigrants, social withdrawal emerged as the only significant variable associated with ethnic bullying victimization. Specifically, the association is quite strong for adolescents born abroad to foreign parents. First generation youths must adapt to a new culture (Berry, 1997, 2005): they have less language skills (Acevedo-Garcìa et al., 2012) and experience more social exclusion in the school context (Plenty and Jonsson, 2017). Furthermore, the stress resulting from being a minority and the process of acculturation are both associated with intra-personal and inter-personal well-being, including bullying victimization (Messinger et al., 2012; Peguero and Jiang, 2014; Vaughn et al., 2014). According to this view, social withdrawal may also be the result of a minority stress condition (Bhugra and Ayonrinde, 2004) and in turn might reinforce the link with victimization. On the other hand, several authors have suggested that social withdrawal may directly lead to peer victimization (Hodges and Perry, 1999; Hubbard et al., 2001). Furthermore Jugert and Titzmann (2017) suggest that immigrant students’ victimization experiences are well predicted by aspects of normative development and interindividual characteristics compared with the acculturative process. Moreover, longitudinal studies showed that lack of confidence in social interactions (Egan and Perry, 1998; Salmivalli and Isaacs, 2005) increase the risk of being bullied.

Therefore, we should consider the necessity to further understand the possible interactive effects of cultural aspects and personality traits in order to better understand successful or risky trajectories of inclusion and acculturation.

The current study has some limitations that need to be addressed. We used cross sectional data to explore the role of acculturation orientation in different groups and this does not allow us to draw causal inferences. Future studies should implement a longitudinal design to examine the acculturative process by taking citizenship status into account in order to confirm and expand our results. While focusing on the citizenship status indicator of the minority group, the present study does not explore the impact of specificities related to some ethnic groups on the relationship between acculturation orientation and ethnic bullying victimization. Some differences could derive from specific immigrants’ characteristics (e.g., country of origin, resources and socio-economic status, ethnicity, and culture, Schwartz et al., 2010). Specifically, the acculturation process is also influenced by the degree of similarity between the heritage and receiving cultures (Rudmin, 2003) and whether the minority and majority groups share the same language (Huntington, 2004). In general, future studies need to understand possible interactions between citizenship status and country of origin in order to confirm that this is a good proxy indicator of ethnic minority groups. Another issue is related to the reference to the “country of origin” in the instruments that could be misleading especially for the group C – adolescents born in Italy with an Italian parent. Further studies need to include a measure related to what participants perceive as country of origin or, at least, control for this factor. Finally, the present study focuses on individual variables (e.g., acculturation orientation and social withdrawal) while looking at a peer group dynamics. It will be valuable to confirm the results and the role of citizenship status when also evaluating the impact of context variables such as racial/ethnic composition both at class and school level, the peers’ prejudices and the interactions of all of these.

## Conclusion

In conclusion, our findings underline the interaction between the individual process of acculturation and citizenship status in peer group dynamics related to ethnic bullying victimization. Previous research underlines the importance of multi-factorial approaches to understand immigrant adolescents and ethnic minority youth victimization dynamics (Jugert and Titzmann, 2017). While immigrant adolescents must be considered firstly adolescents, without further labels, there are immigration-specific factors that are associated with victimization among immigrant youth.

## Data Availability Statement

The datasets generated for this study are available on request to the corresponding author.

## Ethics Statement

The research was carried out in accordance with the Ethic Research Recommendations of the Italian Association of Psychology. It was not approved by an Ethical Research Committee because at that time (2016) it was not yet established at the University of Florence, nor was the approval specifically requested by the Italian Association of Psychology. However, we can state that the research was carried out with the utmost respect for the participants and was in accordance with the Declaration of Helsinki. The schools’, parents’, and students’ active written consents were obtained prior to administrating the questionnaires. Participation was entirely voluntary, confidential and anonymous. The participants were informed that they were free to withdraw from the study at any time.

## Author Contributions

BP and EM contributed conception and design of the study. VZ organized the database. BP performed the statistical analysis. BP and MN wrote the first draft of the manuscript. All authors contributed to manuscript revision, read and approved the submitted version.

## Conflict of Interest

The authors declare that the research was conducted in the absence of any commercial or financial relationships that could be construed as a potential conflict of interest.
